# Non-invasive detection of metastatic papillary thyroid carcinoma after radical surgery using salivary metabolomic biomarkers

**DOI:** 10.20945/2359-4292-2026-0017

**Published:** 2026-02-16

**Authors:** Fei Yu, Jingya Pan, Liuting Zhang, Shaohua Li, Chuan Zhang, Jingjing Fu, Peng Zhou, Jun Wang, Xiaochen Yao, Yudan Ni, Ailing Zhang, Qingle Meng, Rui Yang, Lei Xu, Feng Wang, Jianhua Wang, Liang Shi

**Affiliations:** 1 Department of Nuclear Medicine, Nanjing First Hospital, Nanjing Medical University, Nanjing, China; 2 Department of Thyroid and Breast Surgery, Affiliated Hospital of Integrated Traditional Chinese and Western Medicine of Nanjing University of Chinese Medicine, Nanjing, China; 3 Department of Functional Examination, Nanjing First Hospital, Nanjing Medical University, Nanjing, China

**Keywords:** Metabolomics, metabolite, saliva, metastasis, papillary thyroid carcinoma

## Abstract

**Objective:**

Accurate assessment of metastatic status is crucial for determining radioactive iodine (RAI) dosing in postoperative papillary thyroid carcinoma (PTC) patients. This study aimed to identify unbiased biomarkers in metastatic PTC patients after surgery by applying a metabolomics workflow in saliva samples.

**Materials and methods:**

Saliva samples from 70 postoperative PTC patients (35 metastatic PTC patients in metastasis group and 35 non-metastatic PTC patients in control group) were analyzed using liquid chromatography – mass spectrometry. Orthogonal partial least-squares-discriminant analysis was applied to identify differential metabolites and significant pathways were examined within these metabolites. Receiver operating characteristic curve (ROC) analysis was utilized to further evaluate the diagnostic performance of candidate metabolites.

**Results:**

A total of 119 differential metabolites were identified, with 108 upregulated and 11 downregulated. Pathway analysis revealed 13 significantly dysregulated metabolic pathways in metastatic PTC, including necroptosis, choline metabolism in cancer, sphingolipid signaling, valine, leucine and isoleucine biosynthesis, linoleic acid metabolism and pantothenate and CoA biosynthesis. ROC analysis demonstrated six discriminating biomarkers (5 lipids, 1 amine) that effectively distinguished metastatic from non-metastatic PTC, with all area under the curve values exceeding 0.8. Notably, these metabolites maintained diagnostic performance even in the thyroglobulin antibody-positive subgroup (≥ 4.11 IU/mL) for metastatic screening.

**Conclusion:**

This study demonstrates the potential of salivary biomarkers as a non-invasive diagnostic approach for metastatic PTC to aid the appropriate dosing for RAI therapy. It also offers new insights into the mechanisms of PTC metastasis and potential targets for adjuvant therapy.

## INTRODUCTION

Thyroid cancer (TC) is the most common endocrine malignancy, with its incidence rising steadily annually ^(1,2)^. Among the various types of TC, papillary thyroid carcinoma (PTC) is the most predominant subtype, accounting for approximately 85%-90% of all cases ^(3)^. At the time of diagnosis, around 20%-50% of PTC patients present with lymph node metastases, while 5% have distant metastases ^(4-6)^. Additionally, approximately 10%-30% of patients with PTC develop recurrence or disease progression after initial treatment ^(6)^. Evidently, metastatic PTC patients face substantially elevated risks of disease recurrence and poor clinical outcomes.

The standard treatment for PTC begins with surgical resection, followed by radioactive iodine (RAI) therapy and thyroid-stimulating hormone (TSH) suppression. Patients with persistent lymph node metastasis after initial surgery are typically administered ^131^I at doses of 100-150 mCi, whereas those with distant metastases receive 150-200 mCi ^(6)^. A precise pre-RAI assessment of metastatic status is crucial for determining a patient-specific ^131^I dose, which significantly influences treatment outcomes.

Ultrasonography (US), which is the standard imaging modality for evaluating lymph node metastasis in TC ^(7)^, has been reported to have a diagnostic accuracy of only 72% ^(8)^. Moreover, the computed tomography (CT) manifestations of lung metastases are highly variable, posing significant challenges in distinguishing metastatic nodules from benign lesions ^(9)^. These limitations highlight the insufficiency of relying solely on imaging features for definitive metastasis detection, underscoring the need for integrated diagnostic approaches. Thyroglobulin (Tg) is a widely used biomarker for monitoring PTC, but the prevalence of thyroglobulin antibody (TgAb) can significantly interfere with its accurate measurement ^(10)^. Therefore, there is an urgent need to identify novel biomarkers that can accurately assess metastatic status after radical thyroidectomy.

Metabolomics, a high-throughput technique, quantifies small-molecule metabolites using various specimens like blood, urine, saliva, faeces, tissue, and cell cultures. Cararo Lopes and cols. profiled normal and tumour thyroid tissues from differentiated thyroid carcinoma (DTC) patients, revealing metabolic alterations implicated in energy maintenance and anabolic metabolism in DTC. Furthermore, they identified a panel of six key metabolites – adenosine, ascorbic acid, betaine, guanidoacetic acid, phenylacetic acid, and pyruvate – significantly associated with metastatic PTC ^(11)^. Multiple plasma-based or serum-based metabolomic studies have also revealed distinct metabolite profiles linked to PTC development ^(12-14)^ or metastasis ^(15,16)^. Recently, the use of saliva has attracted much attention in the field of biomedical research because of its advantages of non-invasiveness, ease of collection and storage, and a lack of need for professional operation. Accumulating evidence supports the use of saliva as a promising non-invasive medium for tumour biomarker discovery, with clinical validation in breast cancer ^(17,18)^, oral squamous cell carcinoma ^(19)^, and other malignancies. To date, only one saliva-based metabolomics study has demonstrated that a panel consisting of alanine, valine, proline, and phenylalanine improves the accuracy of early PTC diagnosis ^(20)^. However, existing studies have focused predominantly on preoperative PTC patients, and there remains a lack of research on postoperative patients prior to RAI therapy – particularly regarding the use of salivary metabolic biomarkers to guide RAI dose.

Therefore, to characterize salivary metabolic alterations in postoperative PTC patients scheduled for RAI treatment, we conducted a comprehensive untargeted metabolomic analysis using liquid chromatography-mass spectrometry (LC-MS) to identify potential salivary biomarkers.

## MATERIALS AND METHODS

### Patients

Following total thyroidectomy, patients with PTC were referred to our department for RAI therapy. Approximately 1-2 days before radioiodine administration, we measured serum thyroid hormone, thyroid-stimulating hormone (TSH), stimulated thyroglobulin (s-Tg), and anti-thyroglobulin antibody (TgAb) levels, and conducted neck ultrasonography, chest computed tomography (CT), and whole-body bone scans. The empirical treatment doses of ^131^I were administered under the condition of a serum TSH level of at least 30 mIU/L. Four days after treatment, post-therapeutic ^131^I SPECT/CT was performed for all patients and independently reviewed by two experienced nuclear medicine physicians.

A case-control study was conducted using age-, sex-, and serum TgAb level-matched cohorts. Only patients aged ≥ 18 years with histologically confirmed PTC were included. Postoperative PTC patients presenting with lymph node or distant metastasis were assigned to the metastasis group. Metastatic status was confirmed if at least one of the following criteria was met: (1) Pathological confirmation of metastasis via surgical specimens or fine-needle aspiration biopsy; (2) Imaging evidence (ultrasonography, chest CT scan, whole-body bone scintigraphy, or PET/CT) along with elevated serum Tg and/or TgAb levels; (3) Metastatic lesions identified on post-therapeutic radioiodine whole-body scan after exclusion of physiological uptake. The non-metastasis group included postoperative PTC patients who met all of the following criteria: (1) No evidence of lymph node/distant metastases before and after initial RAI therapy; (2) No biochemical recurrence (defined as suppressed Tg < 0.2 ng/mL or stimulated Tg < 1 ng/mL) or structural disease progression during at least 12 months of follow-up after ablation. All radiographic findings were independently evaluated by two board-certified radiologists with ≥ 10 years of thyroid imaging experience. The study protocol was reviewed and approved by the ethics committee of Nanjing First Hospital (No. KY20240924-10). Informed consent was obtained from all subjects involved in the study.

### Saliva sample collection

All unstimulated saliva samples were collected within a fixed time window (8:30-10:30 a.m.) prior to RAI therapy. Participants were required to avoid eating, drinking, smoking, and oral hygiene procedures for at least 1 hour prior to collection. Additionally, they rinsed their mouths thoroughly with water at least 10 minutes beforehand. During the collection procedure, participants were directed to accumulate saliva in their oral cavities for a minimum of one minute and then expectorate directly into a sterile tube, avoiding inclusion of coughed-up mucus. If the initial attempt did not yield enough saliva, the process was repeated until a minimum volume of 1 mL was obtained. The saliva samples were then centrifuged at 3000 revolutions per minute (rpm) for 10 min at 4 °C. The supernatants were aliquoted and stored at -80 °C until subsequent analysis.

### Untargeted metabolomics analysis of saliva samples

The saliva samples were thawed at 4 °C and 100 µL aliquots were mixed with 400 µL of cold methanol/acetonitrile (1:1, v/v) to remove the protein. The mixture was centrifuged for 20 min (14000 g, 4 °C). The supernatant was dried under vacuum centrifuge. For liquid chromatograph-mass spectrometer (LC-MS) analysis, the samples were reconstituted in 100 µL acetonitrile/water (1:1, v/v) and centrifuged at 14,000 g at 4 °C for 15 min, then the supernatant was injected. Equal volumes of each study sample were combined to create a pooled quality control (QC) sample. All samples were analyzed in the same batch by technicians to avoid batch effects. Continuous sample analysis was conducted in random order, with QC samples inserted into the queue to monitor and evaluate system stability.

Analysis was performed using an UHPLC (Vanquish UHPLC, Thermo) coupled to a Orbitrap Exploris™ 480 in Shanghai Applied Protein Technology Co., Ltd. For Hydrophilic Interaction Liquid Chromatography (HILIC) separation, samples were analyzed using a 2.1 mm × 100 mm ACQUIY UPLC BEH Amide 1.7 µm column (waters, Ireland). In both electrospray ionization (ESI) positive and negative modes, the mobile phase contained A = 25 mM ammonium acetate and 25 mM ammonium hydroxide in water and B = acetonitrile. The gradient was 95% B for 0.5 min and was linearly reduced to 65% in 6.5 min, then reduced to 40% in 1 min and kept for 1min, then increased to 95% in 0.1 min and kept for 2.9 min.

The ESI source conditions were set as follows: Ion Source Gas1 (Gas1) as 50, Ion Source Gas2 (Gas2) as 2, source temperature: 350 °C, IonSpray Voltage Floating (ISVF): +3,500 V/-2,800V. In MS only acquisition, the instrument was set to acquire over the m/z range 70-1200 Da, the resolution was set at 60,000 and the accumulation time was set at 100 ms. In auto MS/MS acquisition, the instrument was set to acquire over the m/z range 70-1,200 Da, the resolution was set at 60,000 and the accumulation time was set at 100 ms, exclude time within 4 s.

### Data processing

The raw MS data were converted to MzXML files using ProteoWizard MSConvert before importing into freely available XCMS software. For peak picking, the following parameters were used: centWave m/z = 10 ppm, peakwidth = c (10, 60), prefilter = c (10, 100). For peak grouping, bw = 5, mzwid = 0.025, minfrac = 0.5 were used. CAMERA (Collection of Algorithms of MEtabolite pRofile Annotation) was used for annotation of isotopes and adducts. In the extracted ion features, only the variables having more than 50% of the nonzero measurement values in at least one group were kept. Compound identification of metabolites was performed by comparing of accuracy m/z value (<10 ppm), and MS/MS spectra with an in-house database established with available authentic standards. Putative metabolite identification was also required to meet level 2 or higher criteria as specified in the Metabolomics Standards Initiative (MSI) guidelines ^(21)^.

### Statistical analysis

For clinical data analysis, continuous variables were compared between two groups using either Student’s t-test (for normally distributed data) or the Wilcoxon rank-sum test (for non-normal distributions). Categorical variables were analyzed using the chi-square test. Statistical significance was defined as *P* < 0.05. All statistical analyses were conducted using R software (version 4.4.0).

In the metabolomic analysis, internal standards were not used for normalization due to the non-targeted nature of the metabolic profiling. Nevertheless, the detected peak areas were log2 normal transformed and then subjected to multivariate analysis using the R package *ropls*. Multivariate analysis included unit variance-scaled principal component analysis (PCA) and orthogonal partial least-squares discriminant analysis (OPLS-DA). Model robustness was evaluated via 7-fold cross-validation and response permutation testing. The contribution of each variable to the classification was assessed based on its variable importance in the projection (VIP) value from the OPLS-DA model. Student’s t test was applied to determine the significance of differences between two groups of independent samples. VIP > 1 and *P* < 0.05 were used to screen significant changed metabolites. KEGG pathway enrichment analysis was performed based on differentially expressed metabolites. The diagnostic performance of metabolites and serum Tg in distinguishing metastatic status in PTC patients was evaluated using receiver operating characteristic (ROC) curve analysis. Correlations between variables were assessed using Pearson correlation analysis.

## RESULTS

### Demographic and clinical data

A total of 70 PTC patients were enrolled and stratified into two groups based on their metastatic status: the metastasis group (35 patients; mean age 41.54 ± 14.39 years, sixteen males and nineteen females) and the non-metastasis group (35 patients; mean age 43.71 ± 13.26 years, sixteen males and nineteen females). No significant differences were observed in age or sex between these two groups (*P* = 0.51 and 1.000, respectively). Serum TgAb levels were comparable between the groups (*P* = 0.87), whereas s-Tg le­vels were significantly elevated in the metastasis group (*P* < 0.001). Further subgroup analysis based on TgAb status demonstrated comparable age, sex, and metastatic site distributions between the TgAb-negative subgroup and the TgAb-positive subgroup. However, the TgAb-positive subgroup had significantly higher TgAb levels and significantly lower Tg levels compared to the TgAb-negative subgroup. More details of these subjects were shown in **Table 1**.

**Table 1.** Characteristics of participants in this study

Variables	Metastasis group (n=35)	Non-metastasis group (n=35)	*P*	TgAb-negative group (n=42)	TgAb-positive group (n=28)	*P*
Age (years)	41.54±14.39	43.71±13.26	0.514	44.29±12.64	40.14±15.23	0.239
Sex						
Male	16 (45.71%)	16 (45.71%)		18 (42.86%)	14 (50.00%)	
Female	19 (54.29%)	19 (54.29%)	1.000	24 (57.14%)	14 (50.00%)	0.732
Tg (ug/l)	84.26 (23.19-292.00)	3.90 (0.36-9.60)	**<0.001**	34.97 (7.09-190.5)	0.85 (0.09-14.48)	**<0.001**
TgAb (IU/ml)	1.94 (0.95-28.05)	2.63 (0.99-8.04)	0.874	1.12 (0.79-1.64)	44.58 (7.51-243.5)	**<0.001**
Metastatic site						
Only cervical lymph node metastasis	22 (62.86%)	/	/	12 (28.57%)	10 (35.71%)	0.713
Distant metastasis	13 (37.14%)	/	/	10 (23.81%)	3 (10.71%)	0.286

Tg: thyroglobulin; TgAb: anti-thyroglobulin antibody.

### Results of sample quality control (QC)

**Figure 1:**
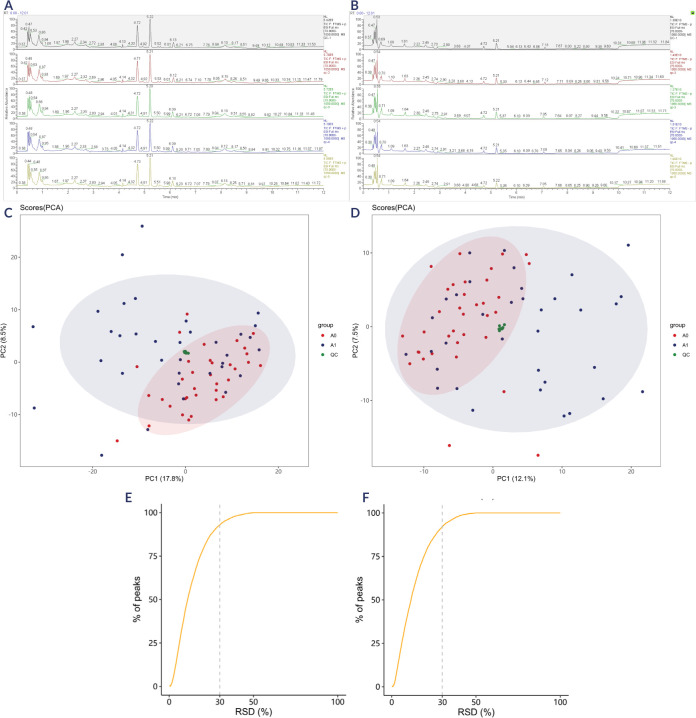
Information of sample quality control. (**A**) Base peak ion chromatograms of samples from each group in positive ion mode. (**B**) Base peak ion chromatograms of samples from each group in negative ion mode. (**C**) PCA analysis of all samples in positive ion mode. metastasis group (A1, blue circles); non-metastasis group (A0, red circle); QC samples (green circles). (**D**) PCA analysis of all samples in negative ion mode. metastasis group (A1, blue circles); non-metastasis group (A0, red circle); QC samples (green circles). (**E**) and distribution of the relative peak area RSD in the QC samples in positive ion mode. (**F**) and distribution of the relative peak area RSD in the QC samples in negative ion mode.

As shown in **Figures 1A** and **1B,** the chromatograms exhibit strong overlap in both positive and negative ion modes. Minimal fluctuations were observed in both retention time and peak response intensity, reflecting that the instrument maintained optimal performance throughout the entire detection process, thereby ensuring stable and reliable signal acquisition. In the PCA of QC samples, the samples were clustered closely together (**Figures 1C** and **1D**, green circles). As indicated in **Figure 1E** and 1F, over 80% of the QC samples exhibited a relative peak area with a relative standard deviation (RSD) ≤ 30%. Overall, the QC results confirmed the high reproducibility and stability of the data in this study.

### Results of metabolites identification and classification

**Figure 2:**
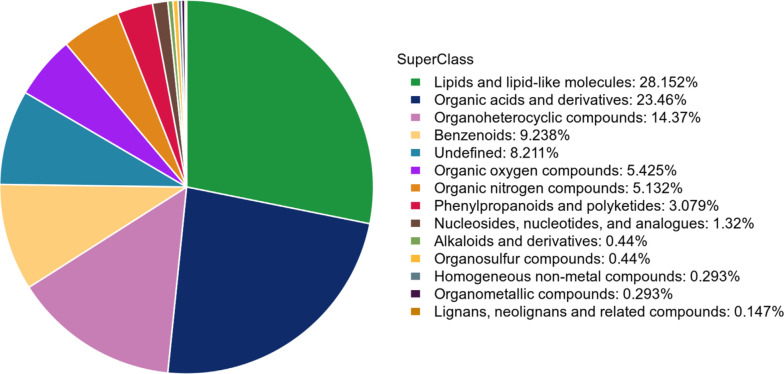
Pie chart of metabolite classification.

After data processing, saliva metabolomic analysis detected 8,810 metabolic features in positive ion mode and 5,371 in negative ion mode across all study samples. Among these, 432 (positive mode) and 250 (negative mode) met level 2 or higher identification criteria according to MSI guidelines. In total, 682 metabolites were definitively identified, with 513 (75.2%) categorized into four major classes: lipids and lipid-like molecules (n = 192, 28.15%), organic acids and derivatives (n = 160, 23.46%), organoheterocyclic compounds (n = 98, 14.37%) and benzenoids (n = 63, 9.24%) (**Figure 2**).

### Identification of differential metabolites

**Figure 3:**
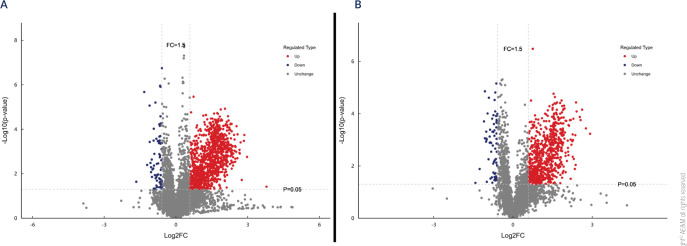
Volcano plot of differential metabolites between the metastasis and non-metastasis group in positive (**A**) and negative (**B**) ion modes.

To identify differential metabolites between the metastasis and non-metastasis group (fold-change ≥ 1.5 or ≤ 0.67 and *P* < 0.05), univariate statistical analysis was performed on all 8810 metabolic features. In positive ion mode, 1109 differential features were identified (1,040 up-regulated, 69 down-regulated); in negative ion mode, 786 were identified (719 up-regulated, 67 down-regulated) (**Figure 3**).

**Figure 4:**
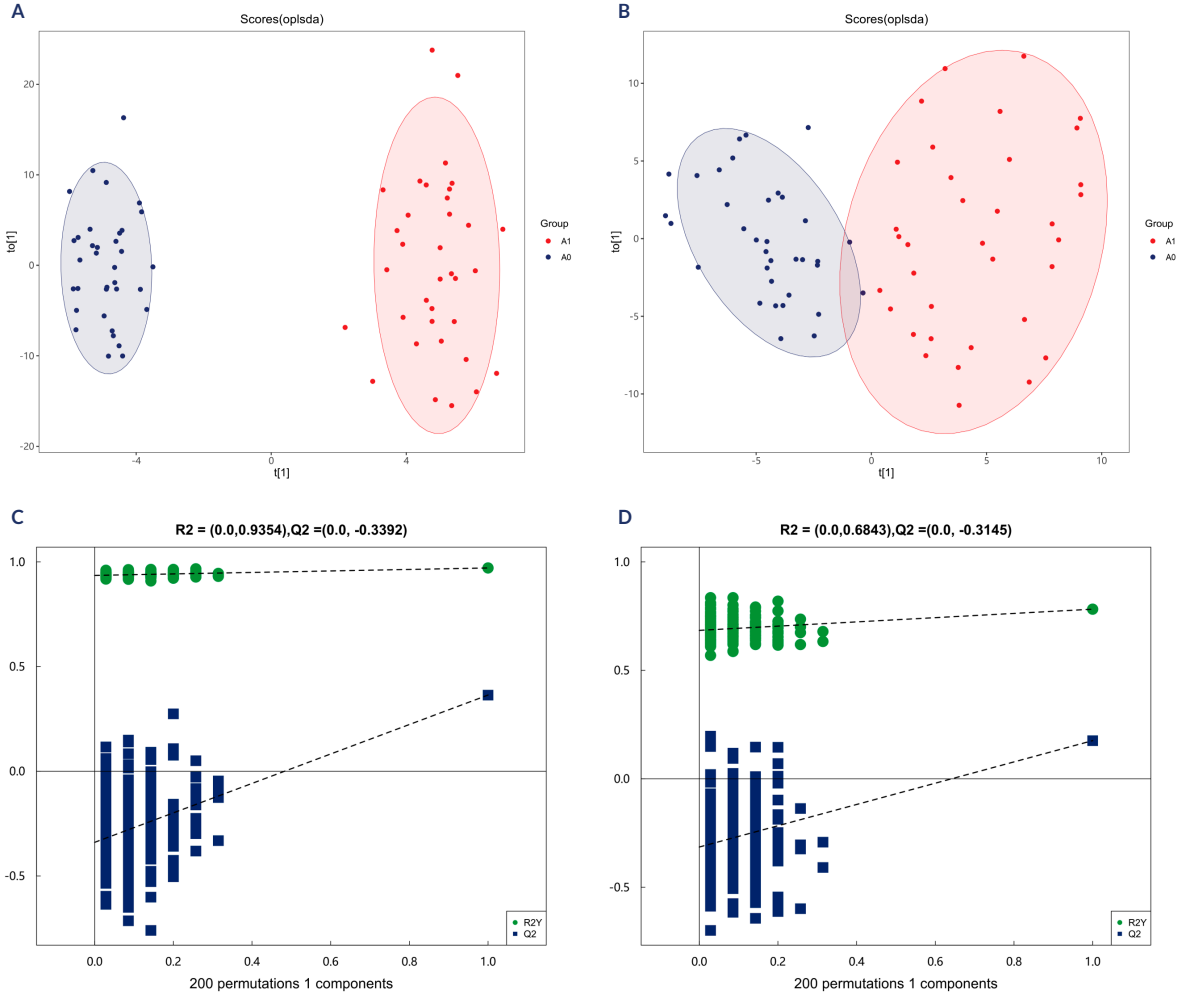
OPLS-DA score plots between the metastasis and non-metastasis group in positive (**A**) and negative (**B**) ion modes. The two rightmost points in the figure are the actual R2Y and Q2 values of the OPLS-DA model, and the remaining points are the R2Y and Q2 values obtained by randomly arranging the samples used (positive (**C)** and negative (**D**) ion modes.

Multivariate statistical analysis was subsequently conducted to obtain a deeper and more comprehensive understanding of the data. PCA was used to obtain an overview of the salivary metabolomic data. As shown in **Figure 1C** and **1D**, the PCA score plot displays a partial but not very obvious separation among the metastasis and non-metastasis groups. We then applied OPLS-DA to examine the metabolomic differences, and the score plot demonstrated a definite separation of samples between the two groups with no overlap (**Figure 4A**, **4B**). Moreover, 200 permutation tests were carried out to verify whether the OPLS-DA model is overfitting, where R2 > 0 and Q2 < 0 indicate a reliable and non-overfit model. The resulting models were considered robust and reliable, with R² and Q² intercepts of 0.935 and -0.339 in positive ion mode, and 0.684 and -0.314 in negative ion mode, confirming the absence of overfitting (**Figures 4C** and **4D**). These results also indicated that the OPLS-DA models exhibit high separating capacity, effectively distinguishing metastatic from non-metastatic PTC patients.

**Figure 5:**
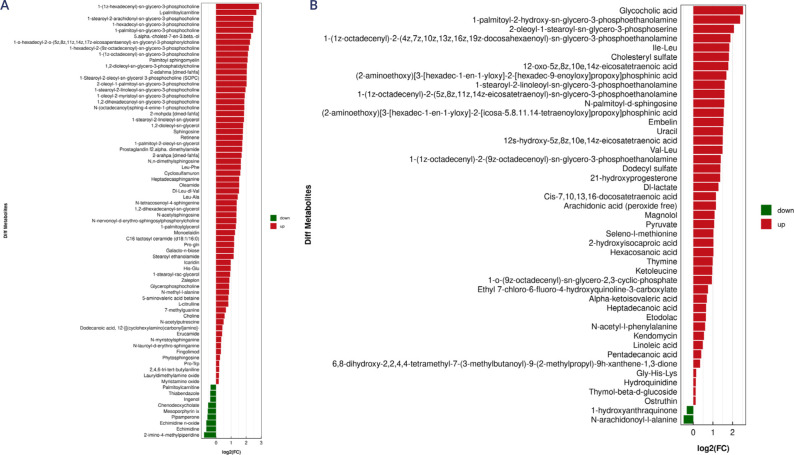
Differential metabolites identified in the positive and negative ion modes in the metastatic group compared to the non-metastatic group.

Differential metabolites between the metastatic group and the metastasis-free group were identified based on the following conditions: 1) VIP ≥ 1 in the OPLS-DA model; 2) *P* < 0.05. A comprehensive analysis revealed a total of 119 differential metabolites, segregated into two ion modes. In positive ion mode, 64 metabolites were upregulated and 9 metabolites were downregulated. In negative ion mode, 44 metabolites were upregulated and 2 metabolites were downregulated (**Figure 5**).

### Metabolic pathway enrichment analysis of differential metabolites

**Figure 6:**
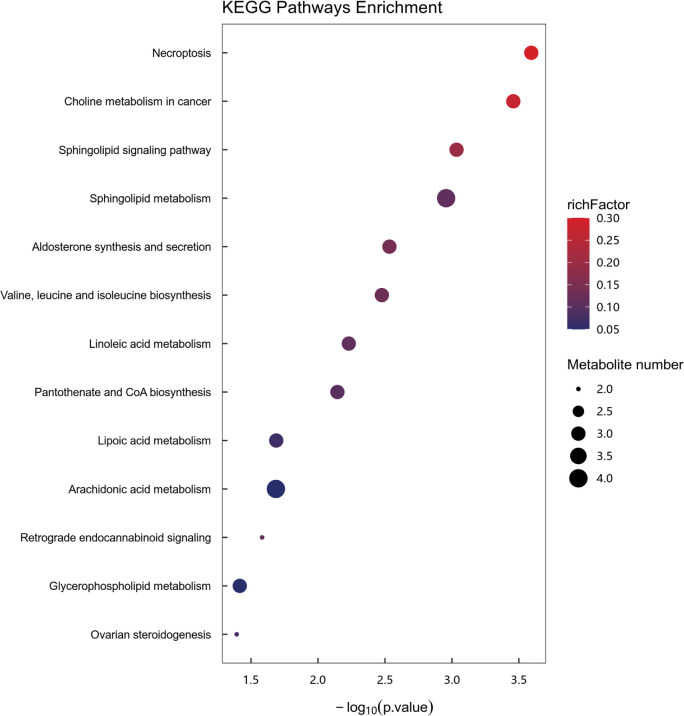
Results of metabolic pathway enrichment analysis of differential metabolites. The color of the dot represents the rich factor and the dot size represents the number of differential metabolites annotated to this pathway.

Enrichment analysis of metabolic pathways was performed using the KEGG database. Pathways with a significance threshold of *P* < 0.05 were considered significantly enriched in differential metabolites. In the current study, metabolic pathway enrichment analysis (depicted in **Figure 6**) was conducted on all 119 differential metabolites, and 13 metabolic pathways that exhibited significant differences between the two groups were identified. These pathways included: necroptosis; choline metabolism in cancer; sphingolipid signaling pathway; sphingolipid metabolism; aldosterone synthesis and secretion; valine, leucine and isoleucine biosynthesis; linoleic acid metabolism; pantothenate and CoA biosynthesis; lipoic acid metabolism; arachidonic acid metabolism; retrograde endocannabinoid signaling; glycerophospholipid metabolism and ovarian steroidogenesis.

### Analysis of the discriminating ability of candidate metabolites

**Figure 7:**
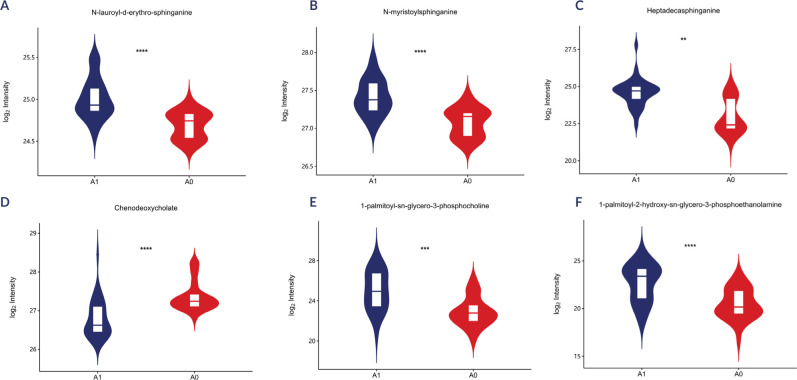
Expression of six discovered metabolites in both groups. Metastasis group (A1, blue); non-metastasis group (A0, red).

**Figure 8:**
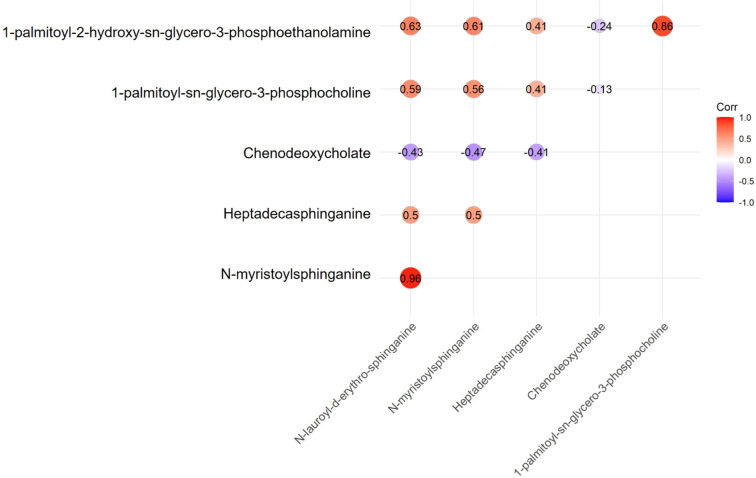
Correlation matrix plots displaying the spearman’s correlation among the six discovered metabolites.

Among the 119 differentially expressed metabolites identified through comprehensive metabolomic profiling, we conducted ROC curve analysis to screen for salivary biomarkers with the potential to discriminate metastatic status in PTC. As shown in **Tables 2-3**, six metabolites, N-lauroyl-d-erythro-sphinganine, N-myristoylsphinganine, heptadecasphinganine, chenodeoxycholate (CDCA), 1-palmitoyl-sn-glycero-3-phosphocholine (PGPC) and 1-palmitoyl-2-hydroxy-sn-glycero-3-phosphoethanolamine (PHGPE) had areas under the curve (AUCs) exceeding 0.8. Quantitative analysis revealed a significantly lower level of CDCA in the metastatic group, in contrast to higher levels of the other five metabolites (**Figure 7**). Furthermore, correlation matrix plots revealed correlations among the six discovered metabolites (**Figure 8**). Of particular interest, CDCA exhibited significant negative correlations with several other metabolites in the network.

**Table 2.** Details of differentially metabolites with AUC > 0.8 in saliva

Name	SuperClass	Level	VIP	Fold change	*P*	AUC^a^	AUC^b^	AUC^c^
N-lauroyl-d-erythro-sphinganine	Lipids and lipid-like molecules	2	1.07	1.25	**<0.001**	0.869	0.873	0.851
N-myristoylsphinganine	Lipids and lipid-like molecules	2	2.61	1.27	**<0.001**	0.862	0.834	0.913
Heptadecasphinganine	Organic nitrogen compounds	2	2.04	2.91	**0.001**	0.859	0.823	0.908
Chenodeoxycholate	Lipids and lipid-like molecules	1	5.17	0.69	**<0.001**	0.834	0.802	0.892
1-palmitoyl-sn-glycero-3-phosphocholine	Lipids and lipid-like molecules	1	3.72	5.47	**<0.001**	0.812	0.782	0.862
1-palmitoyl-2-hydroxy-sn-glycero-3-phosphoethanolamine	Lipids and lipid-like molecules	1	1.45	5.23	**<0.001**	0.802	0.805	0.836

VIP: the variable importance in the projection, obtained from OPLS-DA with a threshold of 1.0; AUC: Area under the curve;

a: AUC calculated in total samples;

b: AUC calculated in samples with negtive TgAb;

c: AUC calculated in samples with postive TgAb.

Level: the confidence level of the identified biomarkers according to the Metabolomics Standards Initiative (MSI) guidelines.

*P: P-*value calculated by *t*-test.

**Table 3.** Quantitative indexes of Tg and six identified biomarkers in all samples

Variables	Class	Cut-off	AUC	Sensitivity	Specificity	PPV	NPV
Tg (ug/l)		21.39	0.827	0.771	0.857	0.844	0.790
N-lauroyl-d-erythro-sphinganine	Sphingolipids	24.85	0.869	0.771	0.829	0.818	0.784
N-myristoylsphinganine	Sphingolipids	27.29	0.862	0.686	0.914	0.889	0.744
Heptadecasphinganine	Amines	23.28	0.859	0.943	0.686	0.750	0.923
Chenodeoxycholate	Steroids and steroid derivatives	26.96	0.834	0.686	0.971	0.960	0.756
1-palmitoyl-sn-glycero-3-phosphocholine	Glycerophospholipids	22.97	0.812	0.886	0.657	0.721	0.852
1-palmitoyl-2-hydroxy-sn-glycero-3-phosphoethanolamine	Glycerophospholipids	22.64	0.802	0.600	0.943	0.913	0.702

AUC: Area under the curve; PPV: Positive Predictive Value; NPV: Negative Predictive Value; Tg: Thyroglobulin.

The cut-off values for the six identified metabolites were determined based on their log2-transformed peak areas.

Serum Tg is the primary clinical tumour biomarker for TC, but its measurement can be interfered by the presence of TgAb. We evaluated the diagnostic performance of serum Tg for metastasis prediction. ROC analysis yielded AUC values of 0.827 across all patients, 0.932 in TgAb-negative samples (TgAb < 4.11 IU/mL), and 0.697 in TgAb-positive samples (TgAb ≥ 4.11 IU/mL) (**Tables 3-4**). We then performed a stratified subgroup analysis based on TgAb levels to systematically evaluate the diagnostic performance of the six candidate metabolites according to TgAb status. Our findings demonstrated that in the TgAb-negative subgroup, all six metabolites maintained robust discriminatory capacity for detecting metastasis. Though these metabolites alone were slightly inferior to Tg, combining Tg with any metabolite improved AUC (notably, the combination of Tg and N-lauroyl-d-erythro-sphinganine achieved an AUC of 0.994). Importantly, in the TgAb-positive subgroup, these metabolites also retained their diagnostic utility for metastatic screening and surpassed serum Tg in performance (**Tables 3-5**).

**Table 4.** Quantitative indexes of Tg and six identified biomarkers across different TgAb status

Variables	TgAb-negtative subgroup						TgAb-positive subgroup					
	Cut-off	AUC	Sensitivity	Specificity	PPV	NPV	Cut-off	AUC	Sensitivity	Specificity	PPV	NPV
Tg (ug/l)	50.50	0.932	0.773	1.000	1.000	0.800	10.70	0.697	0.539	0.933	0.875	0.700
N-lauroyl-d-erythro-sphinganine	24.85	0.873	0.773	0.950	0.944	0.792	24.92	0.851	0.615	1.000	1.000	0.750
N-myristoylsphinganine	27.32	0.834	0.591	0.950	0.929	0.679	27.23	0.913	0.923	0.867	0.857	0.929
Heptadecasphinganine	23.28	0.823	0.909	0.650	0.741	0.867	23.28	0.908	1.000	0.733	0.765	1.000
Chenodeoxycholate	26.97	0.802	0.636	0.950	0.933	0.704	26.96	0.892	0.769	1.000	1.000	0.833
1-palmitoyl-sn-glycero-3-phosphocholine	23.62	0.782	0.773	0.750	0.773	0.750	22.99	0.862	0.923	0.733	0.750	0.917
1-palmitoyl-2-hydroxy-sn-glycero-3-phosphoethanolamine	20.34	0.805	0.864	0.650	0.731	0.813	23.01	0.836	0.769	0.933	0.909	0.824

AUC: Area under the curve; PPV: Positive Predictive Value; NPV: Negative Predictive Value; Tg: Thyroglobulin.

The cut-off values for the six identified metabolites were determined based on their log2-transformed peak areas.

**Table 5.** The diagnostic efficacy of Tg combined with other identified biomarkers in TgAb-negative subgroup

Combined variables	AUC	Sensitivity	Specificity	PPV	NPV
Tg + N-lauroyl-d-erythro-sphinganine	0.994	1.000	0.950	0.957	1.000
Tg + N-myristoylsphinganine	0.949	0.909	0.950	0.952	0.905
Tg + Heptadecasphinganine	0.960	0.773	1.000	1.000	0.800
Tg + Chenodeoxycholate	0.972	0.955	0.950	0.955	0.950
Tg + 1-palmitoyl-sn-glycero-3-phosphocholine	0.972	0.773	1.000	1.000	0.800
Tg + 1-palmitoyl-2-hydroxy-sn-glycero-3-phosphoethanolamine	0.960	0.773	1.000	1.000	0.800

AUC: Area under the curve; PPV: Positive Predictive Value; NPV: Negative Predictive Value; Tg: Thyroglobulin.

## DISCUSSION

Radioactive iodine (RAI) therapy is a cornerstone treatment for patients with DTC following total thyroidectomy and is critical for ablating residual thyroid tissue as well as for treating metastatic disease ^(6)^. Administering an appropriate ^131^I dose optimizes therapeutic efficacy, reduces recurrence, and improves prognosis. Patients with metastatic PTC often require escalated ^131^I activities (150-200 mCi) to achieve effective treatment. Conventional diagnostics (ultrasonography, Tg, CT) may fail to detect occult metastases because of: (1) anatomically complex locations, (2) TgAb interference compromising Tg interpretation, or (3) limited spatial resolution in detecting small pulmonary metastatic lesions. These limitations potentially lead to suboptimal dosing of ^131^I. Therefore, this study employs non-invasive saliva metabolomic profiling in postoperative PTC patients to identify novel predictive biomarkers, thereby enabling personalized ^131^I dosing based on metastatic risk stratification.

Using ROC curve analysis, we identified six salivary biomarkers that demonstrated high diagnostic accuracy in discriminating metastatic from non-metastatic PTC. Among these novel candidates, N-lauroyl-D-erythro-sphinganine, N-myristoylsphinganine, and heptadecasphinganine are sphinganine derivatives that function as components of sphingolipids, which play crucial roles in cell signaling and membrane structure ^(22)^. Notably, bioactive sphingolipids are known to mediate oncogenic processes such as proliferation, migration, and invasion ^(23,24)^, directly supporting their association with metastasis. Similarly, PGPC and PHGPE belong to the phospholipid class, which constitutes fundamental components of cellular membranes and mediate critical biological processes including chemical-energy storage, cellular signaling, and cell-cell interactions. All these processes are pertinent to cellular transformation, cancer progression, and metastasis ^(25)^. In addition to the five identified upregulated lipids in the metastatic group, we also observed significantly downregulated levels of CDCA, a primary bile acid implicated in lipid metabolism. CDCA functions as a high-affinity endogenous ligand for the farnesoid X receptor (FXR) and plays critical regulatory roles through FXR activation to modulate key metabolic pathways ^(26)^. Specifically, CDCA has been shown to suppress gluconeogenesis and de novo lipogenesis, attenuates inflammatory responses, while concomitantly enhancing fatty acid β-oxidation in hepatic and adipose tissues ^(27)^. The regulatory effect of CDCA on lipid metabolism is consistent with the inverse correlation we observed between the CDCA levels and the levels of the five lipids, suggesting that downregulation of CDCA may promote pathogenic lipid accumulation to facilitate metastatic progression. Recent studies have highlighted that CDCA plays a complex role in cancer development and progression, exerting both oncogenic and tumour suppressive effects ^(28-31)^. Our data suggest that perturbations in bile acid-related metabolism occur in metastatic disease. Whether these changes are tumour-suppressive or actionable cannot be inferred from these observational analyses and will require mechanistic and interventional studies.

TgAb compromises the utility of Tg in PTC surveillance by interfering with its assay accuracy. Our TgAb-based subgroup analysis confirmed that Tg effectively predicts metastasis in TgAb-negative PTC patients but has limited predictive value in the TgAb-positive cohort. Notably, the six identified salivary metabolites demonstrated persistent diagnostic robustness for post-operative metastatic surveillance in TgAb-positive PTC patients. This finding may address a critical unmet need in TgAb-interfered clinical monitoring scenarios. Furthermore, the identified novel biomarkers could complement serum Tg levels to enhance the accuracy of metastasis detection in TgAb-negative PTC patients.

Our metabolomic profiling revealed significant disruptions in 13 key metabolic pathways. Necroptosis, a programmed form of necrosis, was identified as the most significantly enriched pathway in our analysis. This pathway has been widely implicated in tumour progression and metastasis across multiple cancer types ^(32,33)^. Equally noteworthy is the aberrant activation of choline metabolism, which is a well-characterized metabolic hallmark of carcinogenesis ^(34)^. This observation is consistent with our previous finding of decreased plasma choline levels in PTC patients compared with both healthy individuals and those with benign nodules ^(35)^. Furthermore, lipid metabolism ^(36)^ and amino acid metabolism ^(37)^ play vital roles in cancer progression and metastasis. In line with these established mechanisms, our results also identified an association between PTC metastasis and alterations in both lipid and amino acid metabolism. Collectively, these findings are biologically plausible and offer novel insights into the molecular mechanisms underlying PTC metastasis.

We also compared our results with those of Cararo Lopes and cols. ^(11)^. Comprehensive analysis revealed that the vast majority of metabolites were upregulated both in DTC versus normal tissue (as reported by Cararo Lopes and cols.) and in metastatic versus non-metastatic samples in saliva (as identified in our study). Notably, metabolites such as choline, pyruvate, and several amino acids, which were reported by Cararo Lopes and cols. to be elevated in DTC tissue, were also consistently upregulated in metastatic PTC in our cohort. Additionally, our results support the importance of anabolic metabolism in PTC progression, corroborating and extending the prior observations of Cararo Lopes and cols. in DTC versus normal tissue. Therefore, our findings suggest that there may be a continuum of metabolic modifications extending from normal thyroid tissue to primary PTC and, further, to metastatic disease.

This study represents the first application of salivary metabolomics in the surveillance of PTC metastasis. Nevertheless, several limitations must be acknowledged. As a single-centre preliminary investigation, this study was limited by a relatively small sample size, the lack of an independent validation cohort, and insufficient mechanistic exploration. Moving forwards, additional work is needed to substantiate the mechanistic insights, such as employing isotope tracing and flux analyses to characterize metabolic rewiring, performing genetic or pharmacological perturbation of candidate metabolic enzymes, and obtaining orthogonal validation in independent clinical cohorts. Future directions should primarily emphasize the application of these metabolite markers in prospective, multi-centre studies to evaluate their clinical utility for monitoring PTC metastasis.

In conclusion, this research provides new insights into the metabolic alterations associated with post-operative PTC metastasis. Six potential salivary biomarkers for the detection of PTC metastasis were successfully identified. This metabolomics investigation further revealed 13 pathways (primarily involved in necroptosis, choline, sphingolipid, and valine, leucine and isoleucine biosynthesis pathways) that are involved in the metastatic progression of PTC, potentially revealing new therapeutic opportunities. These biomarkers and metabolic pathways elucidated in this study open up promising directions for the development of innovative diagnostic methods and therapeutic strategies for PTC patients.

## Data Availability

datasets related to this article will be available upon request to the corresponding author.
